# In Vitro Analysis of Gene and Protein Expression in Primary Limbal Epithelial Cells Exposed to Differentiation-Inducing Medium

**DOI:** 10.3390/biology15080610

**Published:** 2026-04-12

**Authors:** Shweta Suiwal, Virendra Kumar, Tanja Stachon, Priya Katiyar, Fabian N. Fries, Berthold Seitz, Shuailin Li, Shao-Lun Hsu, Shanhe Liu, Swarnali Kundu, Maryam Amini, Sabrina Häcker, Nóra Szentmáry

**Affiliations:** 1 Dr. Rolf M. Schwiete Center for Limbal Stem Cell and Aniridia Research, Saarland University, 66421 Homburg, Germany; virendramicro@gmail.com (V.K.); tanja.stachon@uni-saarland.de (T.S.); priya21katiyar@gmail.com (P.K.); fabian.fries@uks.eu (F.N.F.); shuailinli97@gmail.com (S.L.); arinahsu@gmail.com (S.-L.H.); liushanhe666@gmail.com (S.L.); theswarnalikundu@gmail.com (S.K.); maryam@amini.cloud (M.A.); sabrina.haecker@web.de (S.H.); nora.szentmary@uni-saarland.de (N.S.); 2 Department of Experimental Ophthalmology, Saarland University, 66421 Homburg, Germany; 3 Department of Pharmacology, Sandip Institute of Pharmaceutical Sciences, Nashik 422213, India; 4 Department of Ophthalmology, Saarland University Medical Center, 66421 Homburg, Germany; berthold.seitz@uks.eu

**Keywords:** limbal epithelial cells, KGM3, CnT-2D, differentiation

## Abstract

**Purpose:** To study the time course of the differentiation process and its regulatory networks in primary limbal epithelial cells (pLECs) using serum-free, low calcium Keratocyte growth medium 3 (KGM3) and CnT-2D differentiation medium. **Methods**: pLECs were isolated from corneoscleral rims from healthy donors and cultured in serum-free low calcium (0.06 mM Ca^2+^) KGM3. Differentiation was induced by supplementation with CnT-2D differentiation medium, while control cells were maintained in low-calcium KGM3 medium. Gene and protein expression analyses were performed using qPCR and Western blotting, respectively, at 72 h and at 5, 7, 10, and 14 days post-supplementation to determine the optimal time course of differentiation induction. **Results**: CnT-2D differentiation medium supplementation resulted in a significant upregulation of differentiation-associated markers, including desmoglein 1 (DSG1), paired box domain 6 (PAX6), keratin 3 (KRT3), fatty acid binding protein 5 (FABP5), cellular retinoic acid binding protein 2 (CRABP2), alcohol dehydrogenase 7 (ADH7), aldehyde dehydrogenase 1A1 (ALDH1A1), with the most pronounced changes observed at day 10 post-supplementation (*p* ≤ 0.05). **Conclusions:** CnT-2D differentiation medium effectively initiates differentiation of limbal epithelial cells in vitro. The gradual increase in the expression of key differentiation markers, including DSG1, KRT3, and PAX6, indicates that CnT-2D medium successfully induces differentiation in 2D cultured primary limbal epithelial cells. However, subcellular localization of these markers, epithelial barrier function, and differentiation in 3D models were not assessed and remain to be investigated.

## Simple Summary

Disruptions in differentiation mechanisms can contribute to ocular surface disease, underscoring the need for reliable tools to study epithelial differentiation. To date, only a limited number of systematic studies have examined culture conditions that promote differentiation in primary limbal epithelial cells (pLECs). Under standard conditions, pLECs typically exhibit an undifferentiated phenotype, accompanied by low expression of differentiation markers. In this study, we established optimized culture conditions and a defined time course to induce limbal epithelial cell differentiation in vitro, enabling the analysis of differentiation-associated genes at both transcriptional and translational levels and highlighting the utility of this model for ocular surface research and regenerative applications.

## 1. Introduction

The maintenance of corneal transparency and barrier function depends on the precise differentiation of limbal epithelial stem cells (LESCs). Undifferentiated LESCs reside in the basal epithelium of the limbus and are slow-cycling cells under steady-state conditions, while possessing a high regenerative capacity in response to injury [1,2,3,4]. During differentiation, LESCs give rise to transient amplifying cells (TACs), which subsequently migrate centripetally toward the corneal surface to replenish the corneal epithelium. These cells ultimately differentiate into mature, post-mitotic corneal epithelial cells [5,6]. LESCs are supported by the limbal niche, and the limbal niche provides essential extrinsic cues that initiate and regulate epithelial differentiation in response to environmental and physiological stimuli [6,7]. Disruption of the limbal stem cell niche leads to limbal stem cell deficiency (LSCD), a condition associated with impaired epithelial regeneration, compromised corneal transparency, and, ultimately, vision loss or blindness [2,8,9,10,11]. In addition to niche dysfunction, disturbances in the molecular mechanisms governing corneal epithelial differentiation may also contribute to ocular surface pathologies. PAX6 is a key transcription factor that orchestrates visual system development and plays a critical role in maintaining ocular tissue homeostasis. Its expression at early stages of corneal epithelial cell differentiation highlights a fundamental role in regulating epithelial lineage commitment and maturation [12]. Therefore, pathological alterations of the limbus are also manifested in aniridia-associated keratopathy (AAK), a progressive disorder characterized by corneal opacity, epithelial instability, and neovascularization [13].

Mature corneal epithelial cells are characterized by the expression of specific differentiation markers, including keratins KRT3 and KRT12, as well as the corneal desmosomal protein desmoglein 1 (DSG1). The expression of keratins and desmogleins is widely accepted as a stable readout of corneal epithelial differentiation and maturation [14,15,16,17,18,19]. In parallel, retinoic acid (RA), a biologically active derivative of vitamin A, plays an essential role in regulating epithelial proliferation and differentiation during ocular development. On the ocular surface, RA signaling is tightly regulated by enzymes and transport proteins, including ALDH1A1, ADH7, CRABP2, and FABP5, and disruption of this pathway has been shown to impair epithelial differentiation [14,16,20,21,22,23].

While numerous studies have reported epithelial differentiation across various cell types, differentiation protocols—both in vitro and in vivo—vary considerably, and the most suitable approach to induce differentiation in primary limbal epithelial cells has yet to be clearly defined [14,24,25,26,27]. Therefore, the present study aimed to establish an optimized culture condition and time course for inducing limbal epithelial cell differentiation in vitro, enabling the investigation of differentiation-associated genes at both transcriptional and translational levels. To this end, the expression profiles of corneal (limbal) differentiation markers were analyzed under differentiation-promoting 2D culture conditions and compared with a proliferation-supporting medium used as a control. Given the limited availability of primary limbal epithelial cells, such in vitro models are essential for identifying regulators of epithelial differentiation. In addition, in future studies, these culture conditions may be directly adapted to additional cell models to further investigate, for example, PAX6-associated differentiation mechanisms.

## 2. Materials and Methods

### 2.1. Ethical Considerations

This study was approved by the Ethics Committee of Saarland, Germany (approval no. 21/21; approval date: 15 February 2021) and was conducted in accordance with the principles of the Declaration of Helsinki. Corneoscleral donor rims from 20 healthy donors (mean age 64.05 ± 13.43 years, 60% males) were obtained through the Klaus Faber Center for Corneal Diseases in collaboration with the Lions Eye Bank. Donor demographic characteristics, including age and sex distribution, are summarized in Supplementary Table S1.

### 2.2. Cell Culture

Primary limbal epithelial cell cultures represent a heterogeneous population comprising LESCs, TACs, and differentiated epithelial cells at various stages of differentiation. Therefore, in the present study, the term “primary limbal epithelial cells (pLECs)” is used to describe this mixed population rather than the more restrictive term “limbal progenitor cells”. pLECs were isolated from corneoscleral rims obtained from healthy human donors as described previously [28]. The limbal region was excised using a 1.5 mm biopsy punch, yielding small tissue explants. These explants were incubated overnight at 37 °C in keratinocyte growth medium 3 (KGM3; PromoCell, Heidelberg, Germany) supplemented with 0.5 mg/mL collagenase A (Roche Pharma AG, Basel, Switzerland). On the following day, the digested limbal tissue fragments were pooled and passed through a 20 µm CellTrics^®^ filter (Sysmex, Norderstedt, Germany), thereby removing fibroblast contamination. The filter was subsequently washed with phosphate-buffered saline (PBS), and the retained cell clusters were incubated with 1.5 mL trypsin–EDTA solution (Sigma-Aldrich Merck KGaA, Darmstadt, Germany) to obtain a single-cell suspension. Enzymatic digestion was stopped by adding Dulbecco’s Modified Eagle Medium (DMEM) supplemented with 5% fetal calf serum (FCS) and 100 U/mL penicillin/streptomycin (P/S) (Sigma-Aldrich, Germany).

The resulting cell suspension was centrifuged at 200× *g* for 5 min, and the cell pellet was resuspended in KGM3, a serum-free and bovine pituitary extract (BPE)-free medium (PromoCell, Heidelberg, Germany), supplemented with 0.06 mM Ca^2+^, and 10 U/mL P/S. Cells were seeded into a single well of a 24-well plate and cultured at 37 °C until approximately 90% confluence, with medium changes performed every three days. Upon reaching confluence, pLECs were passaged using 500 µL of trypsin–EDTA. The enzymatic reaction was stopped with 1 mL DMEM, followed by centrifugation at 200× *g* for 5 min. Cells were then resuspended in 3 mL KGM3 medium and replated. Expansion was carried out by passaging cells from one well into three wells or from three wells into six wells of a 6-well plate, depending on proliferation rates. Expanded cells were either used for downstream experiments, including differentiation medium supplementation, or cryopreserved in Cryo-SFM (serum-free medium; PromoCell, Heidelberg, Germany) and stored at −80 °C until further use.

### 2.3. Differentiation of pLECs Using Culture Media

To assess the differentiation potential of pLECs, low-passage cells (passage 2) were seeded into 6-well plates and cultured in two different culture media: serum-free and bovine pituitary extract (BPE)-free Keratinocyte Growth Medium 3 (KGM3; PromoCell, Heidelberg, Germany) and serum-free and BPE-free-CnT Prime 2D Differentiation Medium (CnT-2D; CellnTech, Bern, Switzerland). Initially, cells were cultured in KGM3 to allow for cell attachment. Subsequently, cultures were maintained either in KGM3 or switched to CnT-2D medium and cultured for 72 h or for extended periods of 5, 7, 10, and 14 days.

Culture medium was refreshed every 2–3 days throughout the experimental period. Morphological changes were monitored by light microscopy, and cells were harvested at the indicated time points (72 h, days 5, 7, 10, and 14) for downstream analyses, including gene and protein expression studies.

### 2.4. RNA and Protein Isolation and Quality Control

Total RNA and protein were extracted from KGM3 and CnT-2D medium supplemented with pLECs using the RNA/DNA/Protein Purification Plus Micro Kit (Norgen Biotek Corp., Thorold, ON, Canada; Cat. No. 47700), according to the manufacturer’s instructions. Cells were washed with phosphate-buffered saline (PBS) and lysed in 300 µL SKP lysis buffer containing guanidinium salts to ensure efficient cellular disruption. To enhance lysis and preserve RNA integrity, 3 µL of β-mercaptoethanol was added to the lysis buffer, and samples were incubated at room temperature for 5 min.

Genomic DNA was removed by passing the lysates through the genomic DNA (gDNA) purification column, followed by centrifugation at 5200× *g* for 2 min. The resulting flow-through, containing RNA and protein, was collected for subsequent purification steps. For RNA isolation, 60 µL of 96–100% ethanol was added per 100 µL of flowthrough, and the mixture was applied to the RNA/Protein purification column and centrifuged at 3500× *g* for 2 min. The flow-through from this step was retained for protein purification. The RNA-bound column was washed three times using the provided RNA wash buffer, and total RNA was eluted in 30 µL RNA Elution Buffer by centrifugation at 200× *g* for 2 min. RNA concentration and purity were determined using a NanoDrop 2000 spectrophotometer (Thermo Fisher Scientific, Waltham, MA, USA).

For protein purification, the pH of the RNA column flowthrough was adjusted by adding 100 µL molecular biology-grade water and 8 µL Binding Buffer per 100 µL of sample. The adjusted samples were loaded onto the protein purification column and centrifuged at 5200× *g* for 1 min. The column was washed twice with Protein Wash Solution, and proteins were eluted in 50 µL Protein Elution Buffer. Subsequently, 9.3 µL Protein Neutralizer was added to the eluate, followed by centrifugation at 5200× *g* for 2 min. Protein concentration was quantified using the Bradford assay (Sigma-Aldrich GmbH, Merck KGaA, Darmstadt, Germany).

### 2.5. Reverse Transcription Quantitative Real-Time PCR (RT-qPCR)

Complementary DNA (cDNA) was synthesized from 1 µg of total RNA using oligo(dT) primers, M-MuLV reverse transcriptase reaction buffer, and enzyme mix (OneTaq RT-PCR Kit; New England Biolabs, Ipswich, MA, USA), according to the manufacturer’s instructions. Reverse transcription was performed in a PCR thermocycler (Applied Biosystems, Waltham, MA, USA). For quantitative PCR (qPCR), 1 µL of diluted cDNA (2:3 dilution in nuclease-free water) was used as a template. qPCR reactions were performed in duplicate in 96-well plates using AceQ DNA SYBR Green Master Mix (Vazyme Biotech, Nanjing, China) on a QuantStudio™ 5 Real-Time PCR System (Applied Biosystems, Waltham, MA, USA). Glucuronidase beta (GUSB) and TATA-box binding protein (TBP) served as internal reference genes.

The amplification protocol included an initial denaturation at 95 °C for 2 min, followed by 40 cycles of denaturation at 95 °C for 10 s and annealing/extension at 60 °C for 30 s. Gene expression data were analyzed using QuantStudio™ Design and Analysis Software (v1.x) and exported to Microsoft Excel for further processing. Relative expression levels of target genes were normalized to the geometric mean of GUSB and TBP expression. The stability of the reference genes TBP and GUSB was validated by analyzing ΔCt values and their standard deviations. In our dataset, no relevant differences in mean ΔCt values and low standard deviations were observed for TBP and GUSB between samples of pLECs cultured in either KGM3 or CnT-2D at 72 h and on days 5, 7, 10, and 14. Fold changes in gene expression were calculated using the comparative threshold cycle method (2^−ΔΔCt^). Primer sequences for all genes analyzed are listed in Table 1.

### 2.6. Western Blot

Western blot analysis was performed using 4–12% NuPAGE™ Bis-Tris SDS-PAGE gels (Invitrogen, Waltham, MA, USA). Equal amounts of protein (20 µg) from KGM3 and CnT-2D samples were mixed with Laemmli sample buffer and denatured at 95 °C for 5 min. Samples, along with a dual-color protein molecular weight marker (Bio-Rad Laboratories, Feldkirchen, Germany), were loaded onto the gels. Electrophoresis was carried out at 80 V for 30 min, followed by 100 V for 1 h.

Proteins were transferred onto membranes using a semi-dry blotting system with the Trans-Blot^®^ Turbo Transfer System and Trans-Blot^®^ Turbo Transfer Pack (Bio-Rad, Hercules, CA, USA) for 10 min. Following transfer, membranes were rinsed with distilled water and stained with No-Stain™ Protein Labeling Reagent (Invitrogen, Carlsbad, CA, USA) to visualize total protein. Membranes were then incubated with primary antibodies diluted in blocking buffer/antibody diluent provided with the Western Foxx Kit (BioFroxx GmbH, Einhausen, Germany). Immunoreactive bands were detected using Western Lightning™ Plus ECL chemiluminescent substrate (PerkinElmer Life Sciences, Waltham, MA, USA) and visualized with the iBright™ 1500 Imaging System (Invitrogen, USA). Total protein staining was used for normalization of band signal intensities for each antibody during quantification [28,29]. Band intensities were quantified using Image Studio™ Lite software version 5.2 (LI-COR Biosciences, Lincoln, NE, USA). Antibodies used in this study are listed in Table 2.

### 2.7. Statistical Analysis

Quantitative mRNA and protein expression data were exported as Microsoft Excel files. Fold changes in gene expression were calculated from Ct and ΔΔCt values using the 2^−ΔΔCt^ method. Statistical analyses and data visualization were performed using GraphPad Prism version 10.5.0 (GraphPad Software, San Diego, CA, USA). The normality of data distribution was assessed using the Shapiro–Wilk test. For normally distributed data, an unpaired *t*-test was applied, whereas the Mann–Whitney U test was used for non-normally distributed data. A *p*-value < 0.05 was considered statistically significant. Gene expression levels in KGM3 and CnT-2D from 72 h to days 5, 7, 10, and 14 were analyzed using the nonparametric Kruskal–Wallis test, followed by Dunn’s multiple-comparison post hoc test.

## 3. Results

### 3.1. Elevated Expression of Adhesion (DSG1) and Keratin (KRT3) Markers in pLECs Supplemented with Differentiation Medium (CnT-2D)

**Figure 1:**
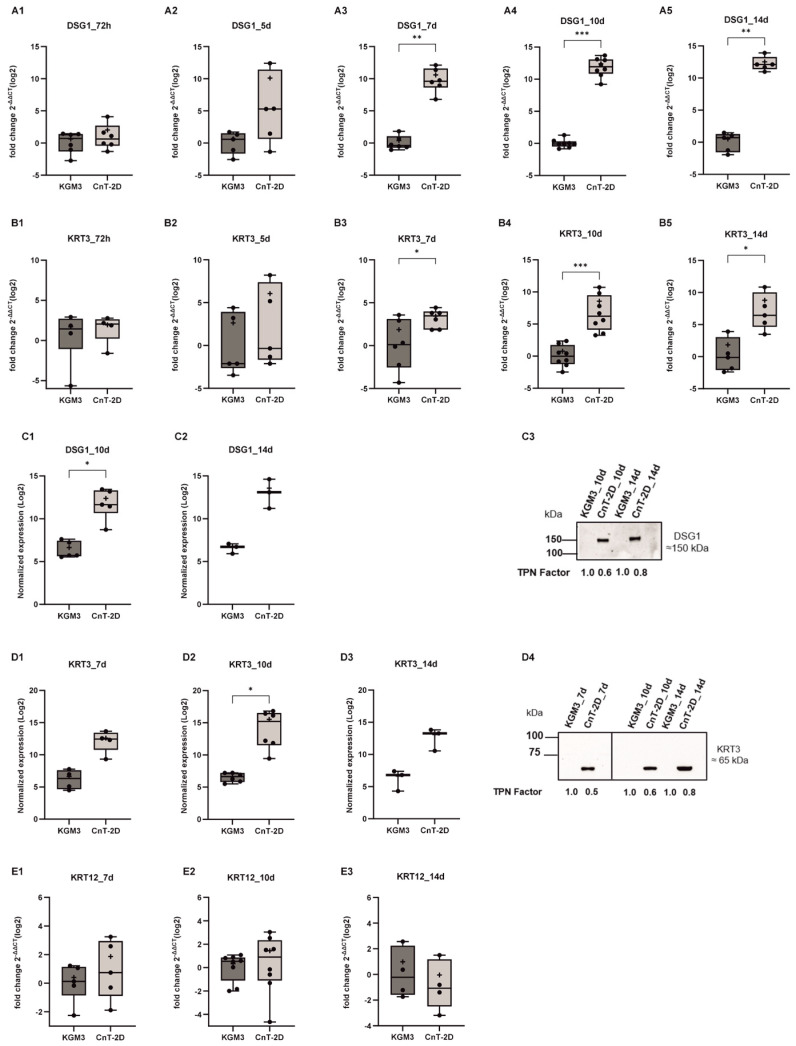
Time course analysis of differentiation markers DSG1 and KRT3 expression in primary limbal epithelial cells (pLECs) under differentiation conditions (from 72 h to 14 days). (**A**,**B**,**E**) *DSG1, KRT3* and *KRT12* mRNA levels in pLECs switched from Keratinocyte Growth Medium (KGM3) to CnT − 2D differentiation medium (CnT 2D) or maintained in KGM3 as control are shown. From day 7 to day 14, *DSG1* and *KRT3* mRNA levels were significantly higher in CnT − 2D medium than in KGM3 (*p* ≤ 0.0079). (**A1**) 72 h (n = 6) *p* = 0.5887, (**A2**) 5 d (n = 5) *p* = 0.1508, (**A3**) 7 d (n = 6) *p* = 0.0022, (**A4**) 10 d (n = 8) *p* = 0.0002, and (**A5**) 14 d (n = 5) *p* = 0.0079 post-switch. (**B1**) 72 h (n = 4) *p* = 0.7861, (**B2**) 5 d (n = 5) *p* = 0.3175, (**B3**) 7 d (n = 6) *p* = 0.0411, (**B4**) 10 d (n = 8) *p* = 0.0002, and (**B5**) 14 d (n = 5) *p* = 0.0159 (**E1**) 7 d (n = 5) *p* = 0.2369, (**E2**) 10 d (n = 8) *p* = 0.1921, (**E3**) (n = 4) *p* = 0.6857. (**C**,**D**) DSG1 and KRT3 protein levels in pLECs switched from KGM3 to CnT − 2D differentiation medium (CnT 2D) or maintained in KGM3 as control. Significant induction of DSG1 and KRT3 protein levels was observed in pLECs supplemented with CnT − 2D for 10 days. (**C1**) 10 d (n = 5) *p* = 0.0332, (**C2**) 14 d (n = 3) *p* = 0.1525, (**D1**) 7 d (n = 4) *p* = 0.0543, (**D2**) 10 d (n = 6) *p* = 0.0453, and (**D3**) 14 d (n = 3) *p* = 0.0881 post-switch. (**C3**) (also see Supplementary Figure S2) and (**D4**) show representative Western blots. The Western blot panel in (**D4**) was assembled by combining lanes from separate blots that were run under identical experimental conditions for presentation purposes. All experimental samples and controls for each comparative analysis were run on the same blot. No data were altered or selectively rearranged (see Supplementary Figure S3). Data are presented as box and whisker plots (min to max). * *p* < 0.05, ** *p* < 0.01, *** *p* < 0.001. Unpaired *t*-test (**B1**,**C1**,**C2**,**D1**–**D3**,**E1**,**E2**); Mann–Whitney U-test (**A1**–**A5**,**B2**–**B5**,**D3**,**E3**).

Gene expression of the differentiation marker DSG1 was analyzed in primary limbal epithelial cells (pLECs) cultured in differentiation medium (CnT-2D) for 72 h, 5, 7, 10, and 14 days, and compared with pLECs maintained in KGM3 medium, which supports an undifferentiated state. *DSG1* mRNA levels remained unchanged after 72 h and 5 days of CnT-2D supplementation (*p* ≤ 0.5887; Figure 1A1,A2). However, *DSG1* mRNA expression was significantly upregulated in cells cultured in CnT-2D medium for 7, 10, and 14 days compared with KGM3-maintained controls (*p* ≤ 0.0022; Figure 1A3–A5).

A similar expression pattern was observed for *KRT3*. mRNA expression of *KRT3* was significantly increased in pLECs cultured in CnT-2D medium for 7, 10, and 14 days compared with controls (*p* ≤ 0.0411; Figure 1B3–B5), whereas no significant changes were detected after 72 h or 5 days of supplementation (*p* ≤ 0.7861; Figure 1B1,B2).

Western blot analysis was performed to assess DSG1 protein level in pLECs cultured in CnT-2D medium for 72 h, 5, 7, 10, and 14 days compared with KGM3 controls. DSG1 protein level was detectable only at days 10 and 14 following CnT-2D supplementation (Figure 1C1,C2). A significant increase in DSG1 protein levels was observed after 10 days of differentiation (*p* = 0.0332; Figure 1C1). Although an increasing trend was evident after 14 days, this did not reach statistical significance (*p* = 0.1525; Figure 1C2).

Similarly, KRT3 protein level was significantly upregulated after 10 days of CnT-2D supplementation (*p* = 0.0453; Figure 1D2). While increased KRT3 protein level was also observed at 7 and 14 days, these changes did not reach statistical significance (*p* ≤ 0.8; Figure 1D1,D3).

In addition to KRT3 expression analysis, KRT12 gene expression was also evaluated. In contrast to KRT3, KRT12 expression did not change significantly in pLECs cultured in CnT-2D medium compared with cells maintained in KGM3 at days 7, 10, and 14 (*p* ≥ 0.1921; Figure 1E1–E3).

*DSG1* and *KRT3* gene expression levels progression over time were assessed in cells cultured with either KGM3 or CnT-2D at 72 h through days 5, 7, 10, and 14 following medium supplementation. In KGM3 medium, no significant differences in *DSG1* or *KRT3* mRNA expression were observed across 72 h, days 5, 7, 10, and 14 (*p* ≥ 0.1593). However, *DSG1* expression increased over time from 72 h to day 10 and day 14, indicating progressive differentiation (*p* ≤ 0.0017), whereas *KRT3* expression remained largely unchanged throughout the observation period (*p* ≥ 0.1555) (Supplementary Figure S1).

### 3.2. Effect of CnT-2D Medium on Stem Cell and Differentiation Markers PAX6 and FABP5

**Figure 2:**
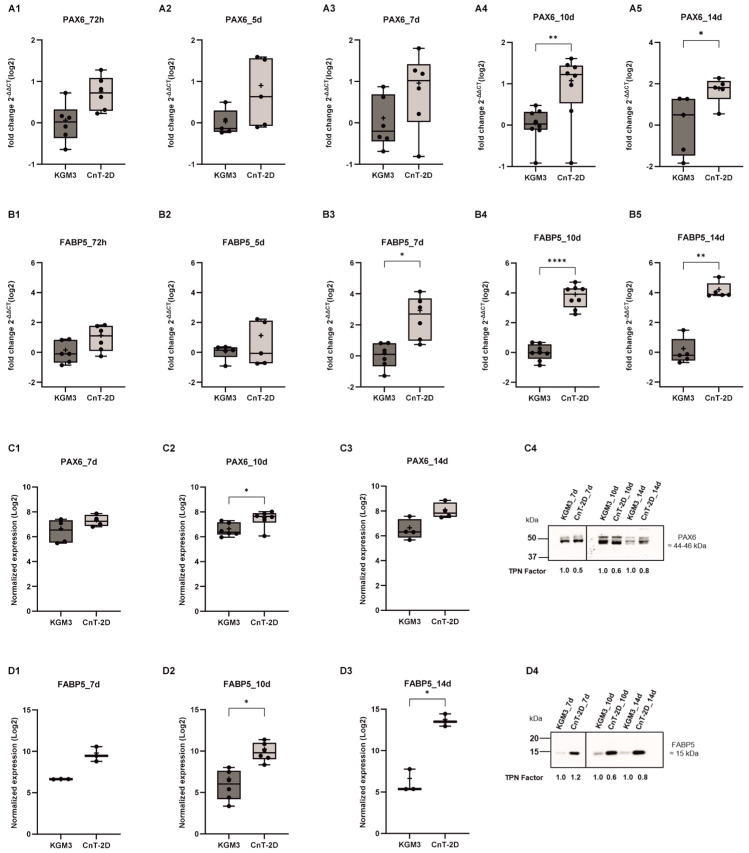
Time course analysis of PAX6 and FABP5 expression in primary limbal epithelial cells (pLECs) under differentiation conditions (from 72 h to 14 days). (**A**,**B**) *PAX6* and *FABP5* mRNA levels in pLECs switched from Keratinocyte Growth Medium (KGM3) to CnT − 2D differentiation medium (CnT 2D) or maintained in KGM3 as control are shown. From day 10, *PAX6* mRNA levels, from day 7, *FABP5* mRNA levels were significantly higher in CnT − 2D medium than in KGM3 (*p* ≤ 0.0079). (**A1**) 72 h (n = 6) *p* = 0.0227, (**A2**) 5 d (n = 5) *p* = 0.1052, (**A3**) 7 d (n = 6) *p* = 0.0919, (**A4**) 10 d (n = 8) *p* = 0.0039, and (**A5**) 14 d (n = 5) *p* = 0.0227 post-switch. (**B1**) 72 h (n = 4) *p* = 0.0680, (**B2**) 5 d (n = 5) *p* > 0.999, (**B3**) 7 d (n = 6) *p* = 0.0288, (**B4**) 10 d (n = 8) *p* < 0.0001, and (**B5**) 14 d (n = 5) *p* = 0.0079 post-switch. (**C**,**D**) PAX6 and FABP5 protein levels in pLECs switched from KGM3 to CnT − 2D differentiation medium (CnT 2D) or maintained in KGM3 as control. Significant induction of PAX6 and FABP5 protein levels was observed in pLECs supplemented with CnT − 2D for 10 days and significant induction of FABP5 protein levels was shown in pLECs supplemented with CnT-2D for 14 days. Blots were probed for PAX6 and TPN (loading control) (**C1**) 7 d (n = 4) *p* = 0.1851, (**C2**) 10 d (n = 6) *p* = 0.0205, (**C3**) 14 d (n = 4) *p* = 0.0520, (**D1**) 7 d (n = 3) *p* = 0.0721, and (**D2**) 10 d (n = 6) *p* = 0.0134 (**D3**) 14 d (n = 3) *p* = 0.0321 post-switch. (**C4**,**D4**) show representative Western Blot. The Western blot panels in (**C4**,**D4**) were assembled by combining lanes from separate blots that were run under identical experimental conditions for presentation purposes. All experimental samples and controls for each comparative analysis were run on the same blot. No data were altered or selectively rearranged (see Supplementary Figures S4 and S5). Data are presented as box and whisker plots (min to max). * *p* < 0.05, ** *p* < 0.01, **** *p* < 0.0001. Unpaired *t*-test (**A1**–**A5**,**B1**,**B3**,**B4**,**C1**–**C3**,**D1**–**D3**); Mann–Whitney U-test (**B2**,**B5**).

Analysis of the stem cell and differentiation markers PAX6 and FABP5 was performed in pLECs cultured in differentiation medium (CnT-2D) for 72 h and for 5, 7, 10, and 14 days. *PAX6* and *FABP5* mRNA expression levels were significantly increased after 10 and 14 days of CnT-2D supplementation compared with pLECs maintained in KGM3 medium (*p* ≤ 0.0227; Figure 2A4,A5,B4,B5). In addition, *FABP5* mRNA expression was already significantly upregulated after 7 days of CnT-2D treatment (*p* = 0.0288; Figure 2B3). In contrast, no significant changes in *PAX6* or *FABP5* transcript levels were detected at earlier time points (72 h and 5 days for both markers, and 7 days for PAX6) compared with KGM3 controls (Figure 2A1–A3,B1,B2).

Protein level analysis by Western blot revealed a significant increase in PAX6 protein levels after 10 days of CnT-2D supplementation (*p* = 0.0205; Figure 2C2). Although an increased trend in PAX6 protein level was observed after 14 days, this difference did not reach statistical significance compared with KGM3 controls (*p* = 0.0520; Figure 2C3). Western blot analysis further demonstrated a significant upregulation of FABP5 protein level after 10 and 14 days of CnT-2D use (Figure 2D2,D3). In contrast, no significant differences in PAX6 or FABP5 protein levels were detected after 7 days of differentiation medium supplementation (*p*≥ 0.1851; Figure 2C1,D1).

*PAX6* and *FABP5* gene expression levels progression over time were assessed over time at 72 h to days 3, 5, 7, 10, and 14 following medium supplementation in cells cultured with either KGM3 or CnT-2D (*p* ≥ 0.1557). FABP5 expression increased progressively during differentiation (*p* ≤ 0.0056), whereas PAX6 expression remained largely unchanged (*p* ≥ 0.1564) (Supplementary Figure S1).

### 3.3. Effect of CnT-2D Medium on Retinoic Acid Pathway Components and Corneal Epithelial Cell Marker

**Figure 3:**
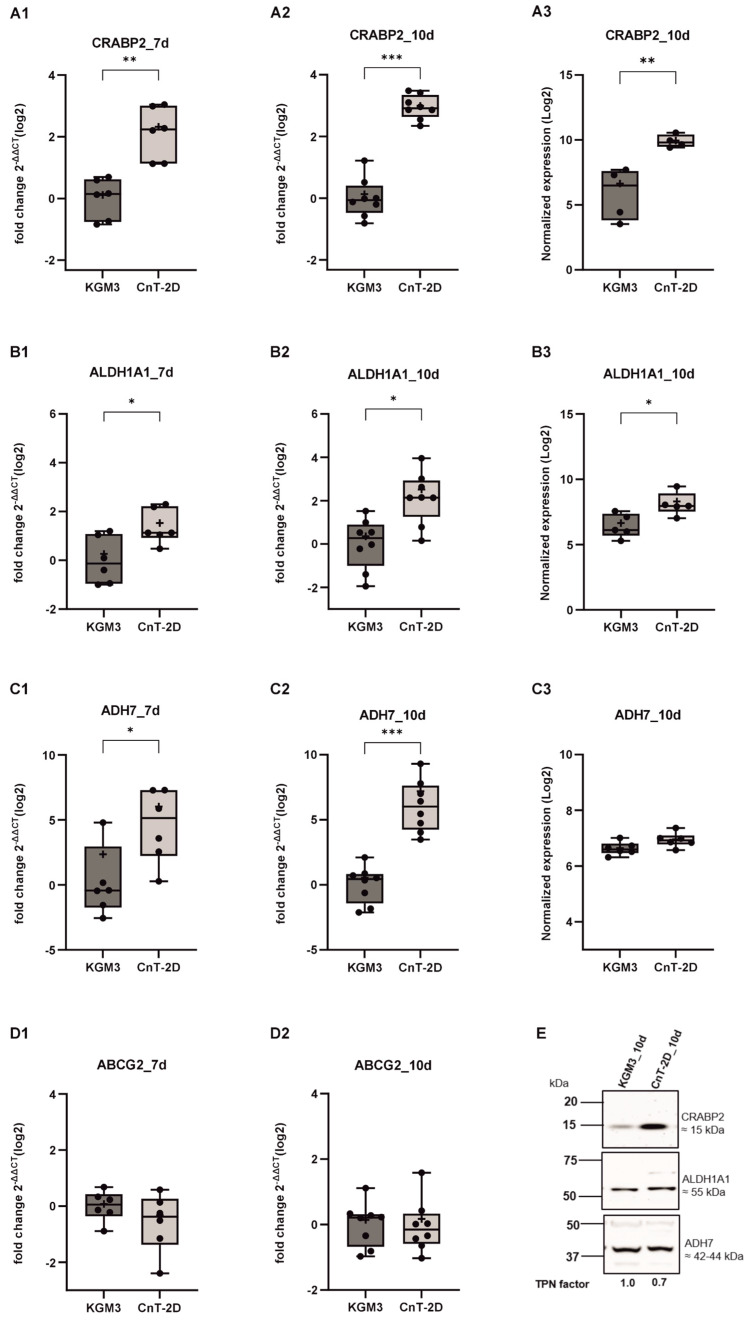
Time course analysis of retinoic acid signalling (RA) pathway components in primary limbal epithelial cells (pLECs) under differentiation conditions at days 7 and 10. (**A**–**D**) *CRABP2* (**A1**,**A2**), *ALDH1A1* (**B1**,**B2**), *ADH7* (**C1**,**C2**) and *ABCG2* (**D1**,**D2**) mRNA levels in pLECs switched from Keratinocyte Growth Medium (KGM3) to CnT−2D differentiation medium (CnT 2D) or maintained in KGM3 as control are shown. Expression fold change for each time point was calculated relative to the respective 7 d or 10 d KGM, normalized to TBP. (**A1**) 7 d (n = 6) *p* = 0.0051, (**A2**) 10 d (n = 8) *p* = 0.0002, (**B1**) 7 d (n = 6) *p* = 0.0327 (**B2**) 10 d (n = 8) *p* = 0.0166, (**C1**) 7 d (n = 6) *p* = 0.0152, (**C2**) 10 d (n = 8) *p* = 0.0002, (**D1**) 7 d (n = 6) *p* = 0.3086, (**D2**) 10 d (n = 8) *p* = 0.6653 post-switch. (**A3**,**B3**,**C3**) show Western blot analyses of CRABP2, ALDH1A1 and ADH7 at day 10, with densitometric quantification normalized to TPN. (**A3**) 10 d *p* = 0.0030 (n = 4) (**B3**) 10 d (n = 5) *p* = 0.0317, (**C3**) 10 d (n = 6) *p* = 0.0642. (**E**) Representative Western blots of CRABP2, ALDH1A1 and ADH7 (also see Supplementary Figures S6–S8). Data are presented as box and whisker plots (min to max). * *p* < 0.05, ** *p* < 0.01, *** *p* < 0.001. Unpaired *t*-test (**A1**,**A3**,**B1**,**C3**,**D1**); Mann–Whitney U-test (**A2**,**B2**,**B3**,**C1**,**C2**,**D2**).

The retinoic acid signaling pathway regulators CRABP2, ADH7, and ALDH1A1 exhibited significantly increased mRNA expression in pLECs cultured in CnT-2D differentiation medium for 7 and 10 days compared with cells maintained in KGM3 medium (*p* ≤ 0.0327; Figure 3A1,A2,B1,B2,C1,C2). After 10 days of CnT-2D supplementation, changes in CRABP2 and ALDH1A1 protein levels paralleled the observed mRNA expression pattern, with a significant upregulation detected in differentiated pLECs (*p* ≤ 0.0317; Figure 3A3,B3). In contrast, changes in ADH7 protein expression did not reach statistical significance (*p* = 0.0642; Figure 3C3).

Additionally, expression of the putative stem cell marker ABCG2, which is typically associated with the undifferentiated state of pLECs, was analysed. The gene expression of ABCG2 remained unchanged in pLECs undergoing CnT-2D–induced differentiation (*p* ≥ 0.3086, Figure 3D1,D2).

### 3.4. Cell Morphology of pLECs Across Different Time Points

**Figure 4:**
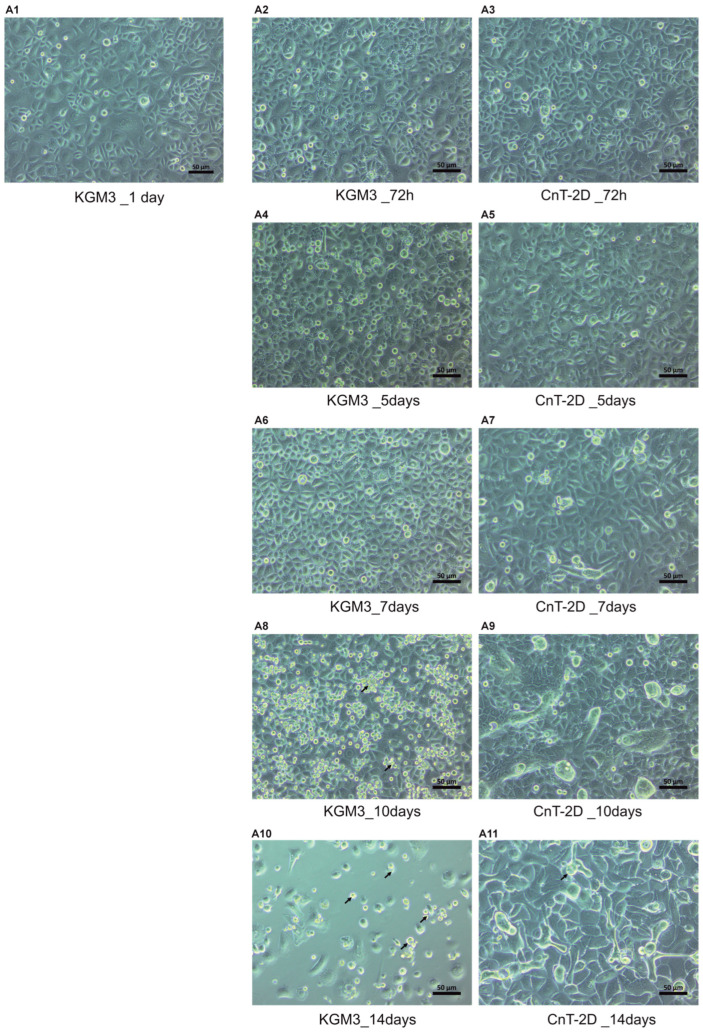
Morphological comparison of primary limbal epithelial cells (pLECs) cultured in Keratinocyte Growth Medium (KGM3) proliferation medium versus CnT − 2D differentiation medium. (**A1**–**A11**) Light microscopy images illustrating the morphological changes in pLECs over a 14-day culture period. pLECs expanded in KGM3 were either maintained in KGM3 medium (control) or switched to CnT 2D differentiation medium. (**A1**,**A2**,**A4**,**A6**,**A8**,**A10**) Cells maintained in KGM3 medium at 24 h, 72 h, and at 5, 7, 10, and 14 days show initial proliferation followed by a progressive decline in cell viability after day 7, characterized by cell rounding, reduced confluency, and signs of cellular stress. (**A3**,**A5**,**A7**,**A9**,**A11**) Cells cultured in CnT − 2D differentiation medium at the corresponding time points exhibit sustained viability accompanied by progressively differentiated cellular morphology. Black arrows indicate dead cells suspended in the culture medium. (Scale bar 50 µm).

pLECs cultured in KGM3 medium, which promotes proliferation and maintains an undifferentiated state, exhibited a typical cobblestone-like morphology [15]. Similarly, at 72 h following CnT-2D differentiation medium supplementation, pLECs retained a cobblestone-like appearance (Figure 4A3). However, after 7 days of CnT-2D treatment, the cells no longer displayed a typical cobblestone-like morphology and a mixture of smaller and larger cells with differentiated-like morphology was observed (Figure 4A7). By day 10 post-supplementation, clear signs of differentiation were evident, characterized by the formation of a uniform monolayer of differentiated cells, in contrast to the monolayer of undifferentiated cells maintained in KGM3 medium (Figure 4A9). At 14 days following CnT-2D supplementation, pLECs appeared differentiated; however, an increased number of dead cells was also observed, potentially indicating reduced cellular survival at this later time point (Figure 4A11).

## 4. Discussion

In the present study, we demonstrated that pLECs undergo differentiation when cultured in CnT-2D differentiation medium and established a time course for pLEC differentiation in vitro. Previous studies have shown that terminal differentiation of epidermal keratinocytes can be induced either by increasing calcium concentrations in the culture medium or by using CnT-2D differentiation medium, which has been successfully applied to promote epithelial differentiation [30,31,32,33]. In contrast, relatively few studies have focused on the differentiation of limbal epithelial cells in vitro. Notably, Ma and Liu reported the effects of calcium on corneal epithelial cell differentiation in culture [27]. Building on these findings, the present study evaluated the differentiation of primary limbal epithelial cells cultured in CnT-2D differentiation medium at multiple time points over a 14-day period, in comparison with KGM3 medium, which supports cellular proliferation.

Our results showed that the mRNA expression of *DSG1* and *KRT3* was significantly increased from day 7 onward following CnT-2D supplementation, indicating that differentiation is initiated after approximately 7 days under these conditions. However, *KRT12* expression remained unchanged. DSG1 is a cell–cell junction protein known to promote epithelial differentiation, particularly in epidermal keratinocytes [34,35]. Similarly, the keratins KRT3 and KRT12 are well-established markers of corneal epithelial differentiation at distinct maturation stages [36,37]. In addition, the significant upregulation of *FABP5*, *CRABP2*, *ADH7*, and *ALDH1A1* further supports the conclusion that CnT-2D medium promotes pLEC differentiation from day 7 onward. FABP5 and components of the retinoic acid signaling pathway, including CRABP2, ADH7, and ALDH1A1, are well-recognized markers of epithelial differentiation, as RA signaling plays a critical role in fine-tuning the balance between cellular proliferation and differentiation [38,39,40].

Nevertheless, protein expression of DSG1, KRT3, FABP5, CRABP2, ADH7, and ALDH1A1 did not show significant upregulation after 7 days of CnT-2D medium supplementation. This discrepancy between mRNA and protein expression following differentiation induction may reflect a temporal delay in protein synthesis during the early adaptive phase of pLECs. The non-linear relationship between mRNA and protein abundance is well documented and can be attributed to multiple post-transcriptional and post-translational regulatory mechanisms, including differences in protein half-life, translation efficiency, and degradation rates [41,42,43,44]. Consequently, changes at the transcript level alone may be insufficient to immediately detect corresponding changes in protein expression. These findings suggest that a 7-day exposure to CnT-2D medium is insufficient to induce full differentiation of primary limbal epithelial cells in vitro.

Considering the observed temporal delay in protein expression at day 7, differentiation of pLECs was further evaluated at 10 and 14 days following CnT-2D supplementation. Notably, both mRNA and protein expression levels of the differentiation markers DSG1 and KRT3 were significantly increased at day 10 post-supplementation. Importantly, DSG1 protein expression was detectable only in cells cultured in CnT-2D medium and was completely absent in cells maintained in the proliferative KGM3 medium, indicating that its induction is specific to differentiation-promoting conditions. This observation suggests that the pronounced increase in *DSG1* mRNA following CnT-2D supplementation translates into detectable protein expression only after prolonged differentiation. In contrast, the expression level of the corneal marker KRT12 remained unchanged at day 10 after supplementation. Mature corneal epithelial cells are characterized by high expression levels of both KRT12 and KRT3. The increased KRT3 expression together with unchanged KRT12 expression suggests that CnT-2D medium did not trigger terminal differentiation of pLECs at day 10 after supplementation.

Moreover, the mRNA expression level of the putative stem cell marker ABCG2 [45] remained unchanged at day 10 following CnT-2D medium supplementation. The stable ABCG2 expression indicates that, during CnT-2D–directed differentiation of pLECs, a proportion of undifferentiated cells is still present in the culture, further supporting the hypothesis that CnT-2D medium does not induce terminal differentiation under these conditions.

In addition, the expression of PAX6, an early differentiation marker of corneal epithelial cells [12], was assessed at day 10 post-supplementation. Both PAX6 mRNA and protein levels were significantly elevated in response to CnT-2D treatment, further supporting the conclusion that pLECs undergo differentiation after approximately 10 days in differentiation medium. The absence of DSG1 and KRT3 protein expression in cells maintained in KGM3 proliferation medium confirms that the observed differentiation-associated changes are specific to CnT-2D-induced differentiation and are not attributable to prolonged culture duration or increased cell confluence.

Moreover, mRNA expression of key retinoic acid signaling components, including *FABP5*, *CRABP2*, *ALDH1A1*, and *ADH7*, was significantly upregulated following CnT-2D differentiation medium supplementation. In contrast, increased protein expression was observed for FABP5, CRABP2 and ALDH1A1. Given that RA signaling components collectively regulate epithelial proliferation and differentiation, this discrepancy between transcriptional and translational regulation of ADH7 in response to CnT-2D medium likely reflects post-translational buffering mechanisms. Post-translational buffering refers to a regulatory phenomenon in which protein levels of upstream pathway components are maintained at a steady state despite changes in corresponding mRNA levels, thereby compensating for alterations occurring at downstream levels of the pathway [46,47,48,49,50]. In addition, the decoupling of protein abundance from mRNA expression may also result from differences in protein stability, including prolonged protein half-life or enhanced protein degradation [46].

At day 14 following CnT-2D supplementation, mRNA expression of *DSG1*, *KRT3*, *PAX6* and *FABP5* remained significantly elevated. However, with the exception of FABP5, whose mRNA and protein expression were both significantly increased, the remaining upregulated transcripts did not translate into corresponding increases at the protein level. These findings suggest that while CnT-2D medium induces differentiation of primary limbal epithelial cells as early as day 7 and maintains a differentiated state through day 14, the most robust and coordinated induction of differentiation, evident at both the mRNA and protein levels, occurs at approximately day 10 following medium supplementation.

The morphology of pLECs cultured in CnT-2D differentiation medium was markedly different from that of cells maintained in KGM3 medium. Under proliferative conditions in KGM3, pLECs displayed a typical small, cobblestone-like morphology with a high nucleus-to-cytoplasm ratio, as extensively described in previous studies [14,15,51]. Notably, up to day 5 following CnT-2D supplementation, pLECs exhibited a morphology comparable to that of cells cultured in KGM3, further indicating that differentiation was not yet initiated during this early period. In contrast, by day 7, pLECs cultured in CnT-2D medium began to display irregular morphology, and by day 10, cultures consisted of a heterogeneous population of small and larger cells, consistent with progressing differentiation. Meanwhile, pLECs maintained in KGM3 medium showed reduced viability and seemed to show signs of cell death after day 10. By day 14, cellular morphology became inconsistent in both KGM3- and CnT-2D-treated cultures (Figure 4). Taken together, the combined evaluation of cellular morphology and mRNA and protein expression profiles indicates that pLECs exhibit clear signs of differentiation at day 10 following CnT-2D medium supplementation.

Through this study, we address a critical gap by defining a culture condition, CnT-2D differentiation medium supplementation, that effectively induces differentiation in primary limbal epithelial cells. Importantly, this culture system may be directly combined with PAX6 siRNA-mediated knockdown models to further investigate the role of PAX6 in regulating limbal epithelial cell differentiation.

Although our study provides a defined and technically straightforward approach to induce differentiation of pLECs in a controlled and reproducible manner in in vitro cultures, several limitations should be considered. The differentiation protocol was established using CnT-2D medium in a two-dimensional in vitro culture system, which does not fully recapitulate the highly specialized three-dimensional (3D) limbal niche present in vivo. Consequently, spatial organization and cell–matrix interactions that regulate epithelial homeostasis in vivo are not represented in this model. Future studies using physiologically relevant 3D systems, such as stratified air–liquid interface cultures or organoid models supported by media such as CnT-Prime epithelial 3D medium [52,53], may further validate and extend the differentiation processes described here.

Our study focused on establishing a detailed time course gene expression profile during induced differentiation, as the relative expression level of differentiation markers in pLECs cultured in KSFM medium is relatively low [16]. The approach described in this study enables the induction of differentiation under defined conditions, thereby facilitating the detection of differentiation marker genes in pLECs. However, a key limitation of our study is the limited availability of primary samples, which prevented us from extending the analysis beyond the expression profiling of differentiation markers. The subcellular distribution of the differentiation markers was not assessed in the current study, limiting conclusions regarding their spatial organization within the cells. Functional assays assessing epithelial barrier integrity (e.g., transepithelial electrical resistance or permeability measurements) or cellular apoptosis could not be performed due to the limited availability of primary human donor tissue required to establish sufficiently large parallel pLEC cultures. Future studies incorporating such functional assays will be important to further validate the barrier-forming capacity of differentiated pLECs.

Notwithstanding these limitations, our study could serve as a crucial baseline for preclinical drug testing. Dorot et.al. and Oved et.al. reported rescue of PAX6 haploinsufficiency with antipsychotropic drugs duloxetine and ritanserin in mutant undifferentiated limbal epithelial cells [54,55]. The described procedure could be easily translated to investigate the effect of these drugs in differentiated PAX6 knockdown cell models. Furthermore, the simple, reliable described approach is forecasting the possibility of future modification in line with good manufacturing practices in order to support therapeutic strategies aimed at ocular diseases.

## 5. Conclusions

In conclusion, CnT-2D differentiation medium efficiently promotes the onset of limbal epithelial cell differentiation in vitro. The progressive upregulation of key differentiation markers, such as DSG1, KRT3, and PAX6, demonstrates its capacity to drive differentiation in primary limbal epithelial cells. The applied culture conditions promote the expression of differentiation-associated markers in a two-dimensional culture system. However, subcellular localization of differentiation markers, functional properties of the epithelial barrier, such as transepithelial electrical resistance (TEER) and permeability, are not assessed and therefore remain to be determined. Future studies should also evaluate differentiation in three-dimensional models.

## Figures and Tables

**Table 1:** Primer pairs used for qPCR.

Referred as	Qiagen Cat. No	Amplicon Size (bp)
ABCG2	QT00073206	114
ADH7	QT00000217	85
ALDH1A1	QT00013286	97
CRABP2	QT00063434	140
DSG1	QT00001617	96
FABP5	QT00225561	97
GUSB	QT00046046	96
KRT3	QT00050365	118
KRT12	QT00011949	104
PAX6	QT00071169	113
TBP	QT00000721	132

**Table 2:** Antibodies used for Western blot analysis.

Antibody	Catalog Number	Dilution
ADH7 rabbit polyclonal antibody	#PA5–98484, Thermo Fischer Scientific, Waltham, MA, USA	1:1000
ALDH1A1 (H-4): mouse monoclonal antibody	sc-374076, Santa Cruz Biotechnology, Santa Cruz, CA, USA	1:1000
CRABP2 mouse monoclonal antibody	#66468–1-lg, Thermo Fischer Scientific, Waltham, MA, USA	1:1000
FABP5 rabbit polyclonal antibody	#12348–1-AP, Proteintech, Rosemont, IL, USA	1:1000
PAX6 rabbit polyclonal antibody	#AB2237, Merk, Darmstadt, Germany	1:1000
KRT3 mouse monoclonal antibody	CBL218, Merk, Darmstadt, Germany	1:500
DSG1 mouse monoclonal antibody	Sc-59904, Santa Cruz Biotechnology, Santa Cruz, CA, USA	1:200

## Data Availability

All data generated during this study are included in the article and its Supplementary Information Files. Further inquiries should be addressed to Shweta Suiwal and Nóra Szentmáry.

## Supplementary Materials

The following supporting information can be downloaded at: https://www.mdpi.com/article/10.3390/biology15080610/s1, Table S1: Donor information; Figure S1: qPCR; Figures S2–S8: WB.

## Abbreviations

The following abbreviations are used in this manuscript:

ABCG2	ATP-binding cassette, subfamily G, member 2
ADH7	alcohol dehydrogenase 7
ALDH1A1	aldehyde dehydrogenase 1A1
CRABP2	cellular retinoic acid binding protein 2
CnT-2D	CnT prime 2D differentiation medium
DSG1	desmoglein 1
FABP5	fatty acid binding protein 5
KGM3	Keratinocyte growth medium 3
KRT3	keratin 3
KRT12	keratin 12
pLECs	Primary Limbal epithelial cells
PAX6	paired box domain 6
