# Supplementary material for: In Vitro Analysis of Gene and Protein Expression in Primary Limbal Epithelial Cells Exposed to Differentiation-Inducing Medium
Source: Biology (Basel). 2026 Apr 12;15(8):610. doi: 10.3390/biology15080610 (PMC13113695; doi:10.3390/biology15080610)
Supplement: Supplementary file 1 [file biology-15-00610-s001.zip › biology-4171779-supplementary.pdf]

**Supplementary Table S1. Characteristics of donors included in the study.**

| Donors | Age | Sex |
|---|---|---|
| 1 | 45 | M |
| 2 | 75 | M |
| 3 | 60 | F |
| 4 | 76 | F |
| 5 | 70 | M |
| 6 | n.a. | n.a. |
| 7 | 66 | M |
| 8 | 40 | n.a. |
| 9 | 78 | M |
| 10 | 68 | n.a, |
| 11 | 84 | F |
| 12 | 69 | F |
| 13 | 41 | n.a. |
| 14 | 46 | F |
| 15 | 67 | M |
| 16 | n.a. | n.a. |
| 17 | 75 | M |
| 18 | 56 | M |
| 19 | 62 | F |
| 20 | 75 | M |

n.a.: not available; M: male, F: female

**Supplementary Figure S1.**

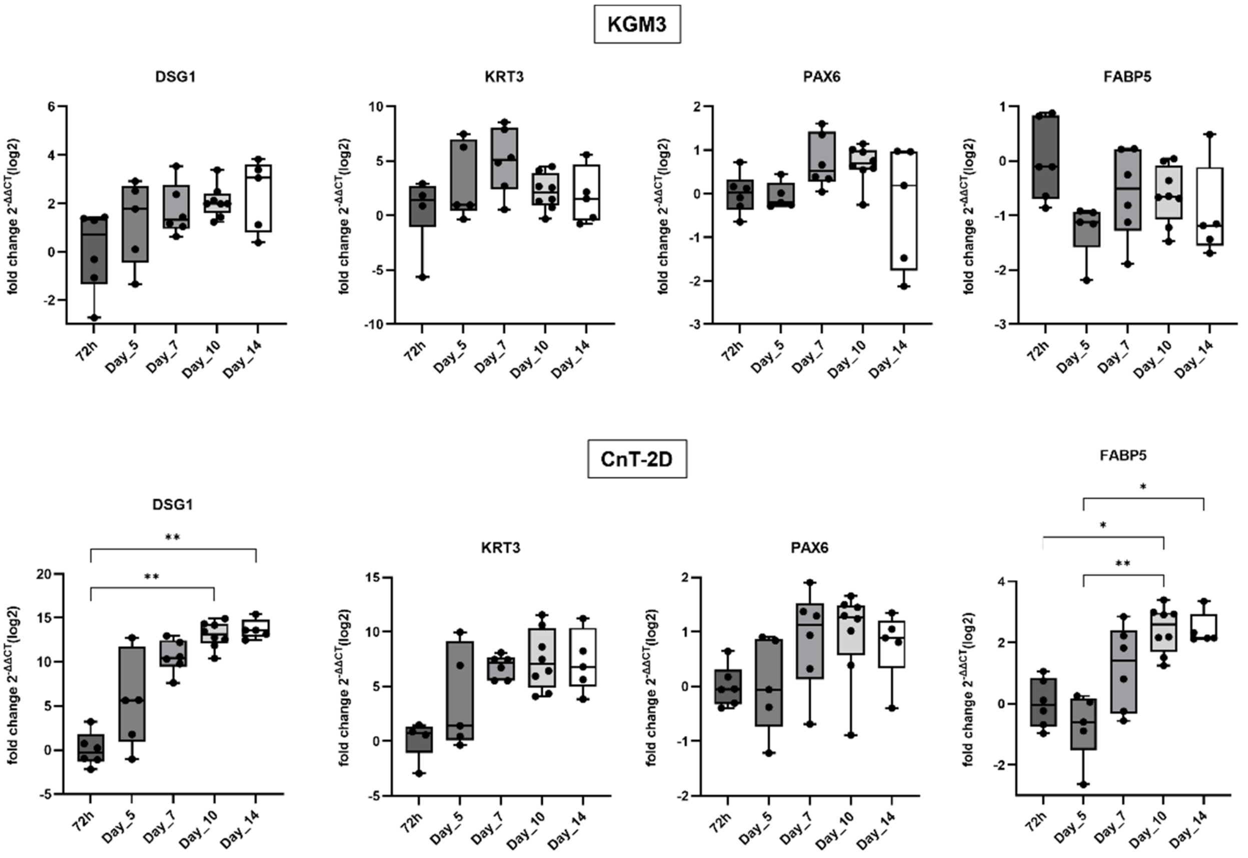


**Supplementary Figure S1.** Time-course analysis of DSG1, KRT3, PAX6, and FABP5 mRNA expression in primary limbal epithelial cells (pLECs) cultured in KGM3 and CnT-2D media. Data are presented as box-and-whisker plots (minimum to maximum). Gene expression levels over time at 72h to days 5, 7, 10, and 14 were analyzed using the nonparametric Kruskal–Wallis test, followed by Dunn's multiple-comparison post hoc test. *$p < 0.05$, **$p < 0.01$, ***$p < 0.001$, ***$p < 0.0001$.

**Supplementary Figure S2.**

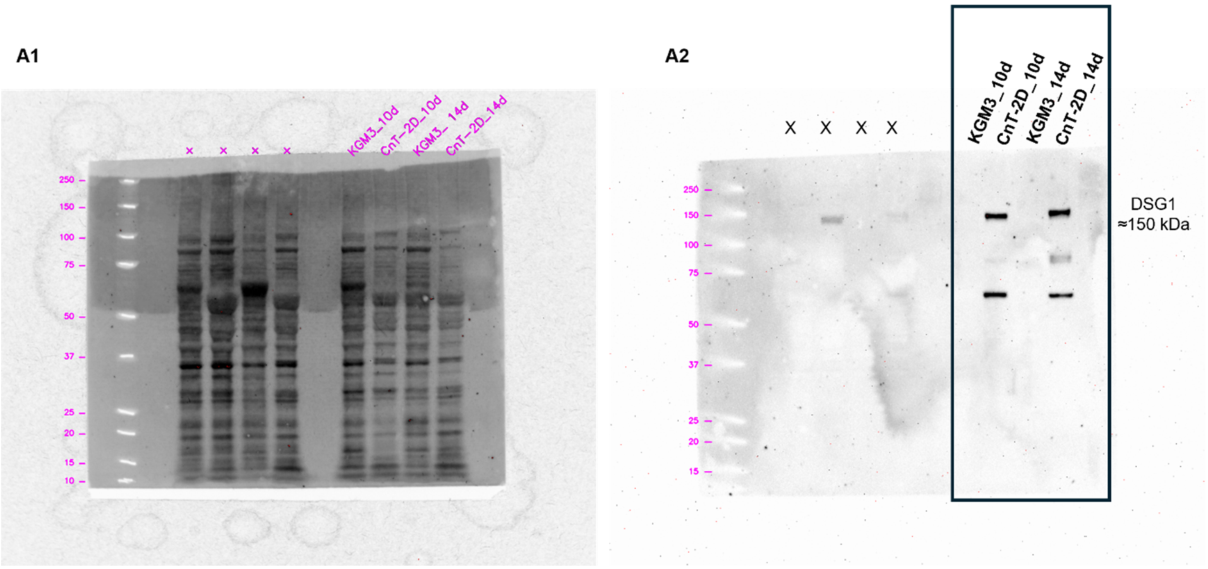


**Supplementary Figure S2. DSG1 Western blot images. (A1)** Total protein normalization (TPN) was performed using the Invitrogen™ No-Stain™ Protein Labeling Reagent to ensure equal protein loading. **(A2)** DSG1 protein bands were detected at the expected molecular weight of approximately 150 kDa in primary limbal epithelial cells (pLECs) switched from Keratinocyte Growth Medium (KGM3) to CnT-2D differentiation medium (CnT-2D), or maintained in KGM3, for 10 and 14 days.

**Supplementary Figure S3.**

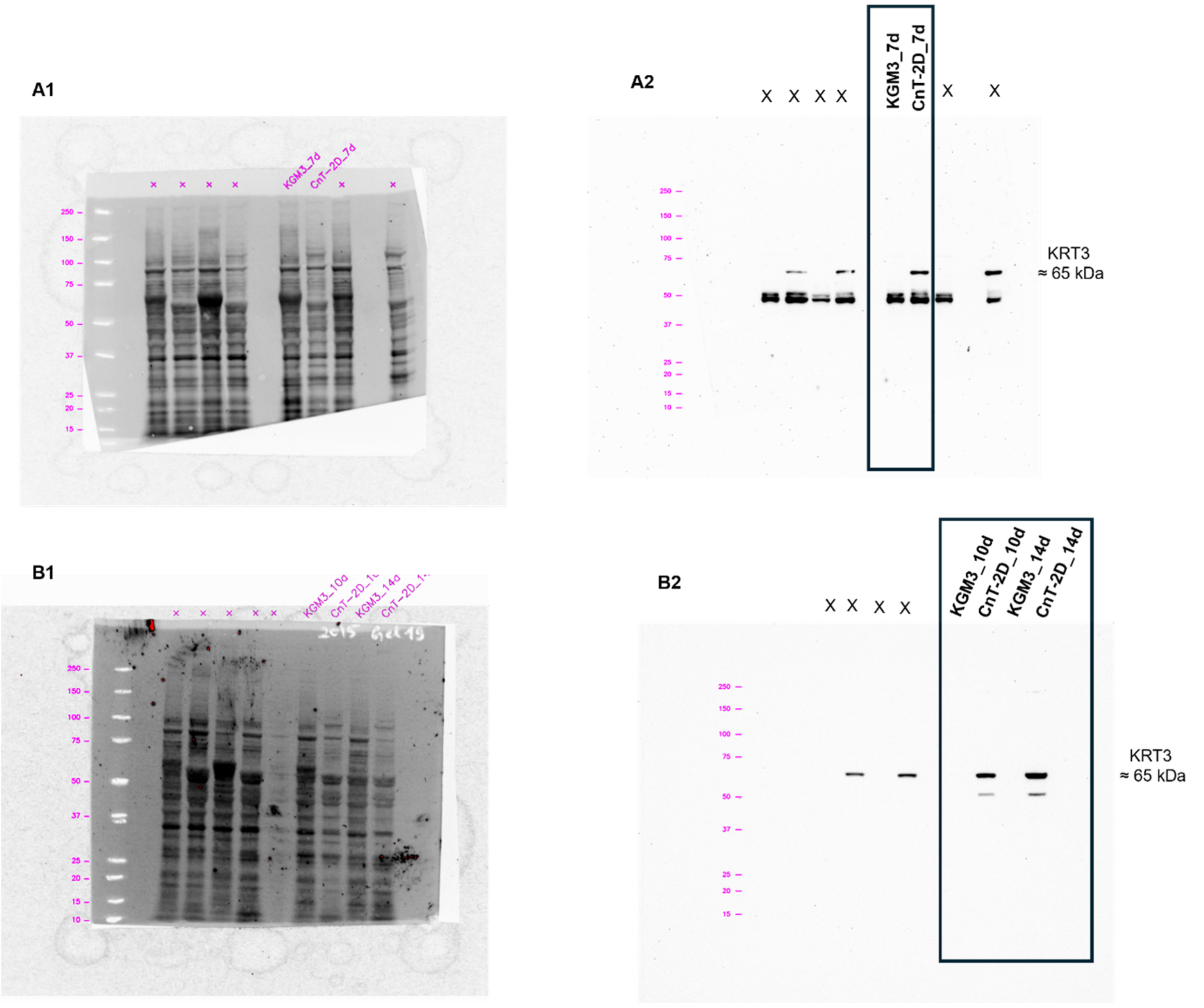


**Supplementary Figure S3. KRT3 Western blot images. (A1, B1)** Total protein normalization (TPN) was performed using the Invitrogen™ No-Stain™ Protein Labeling Reagent to ensure equal protein loading. **(A2, B2)** KRT3 protein bands were detected at the expected molecular weight of approximately 65 kDa in primary limbal epithelial cells (pLECs) switched from Keratinocyte Growth Medium (KGM3) to CnT-2D differentiation medium (CnT-2D), or maintained in KGM3, for 7 days **(A2)** and for 10 and 14 days **(B2).**

**Supplementary Figure S4.**

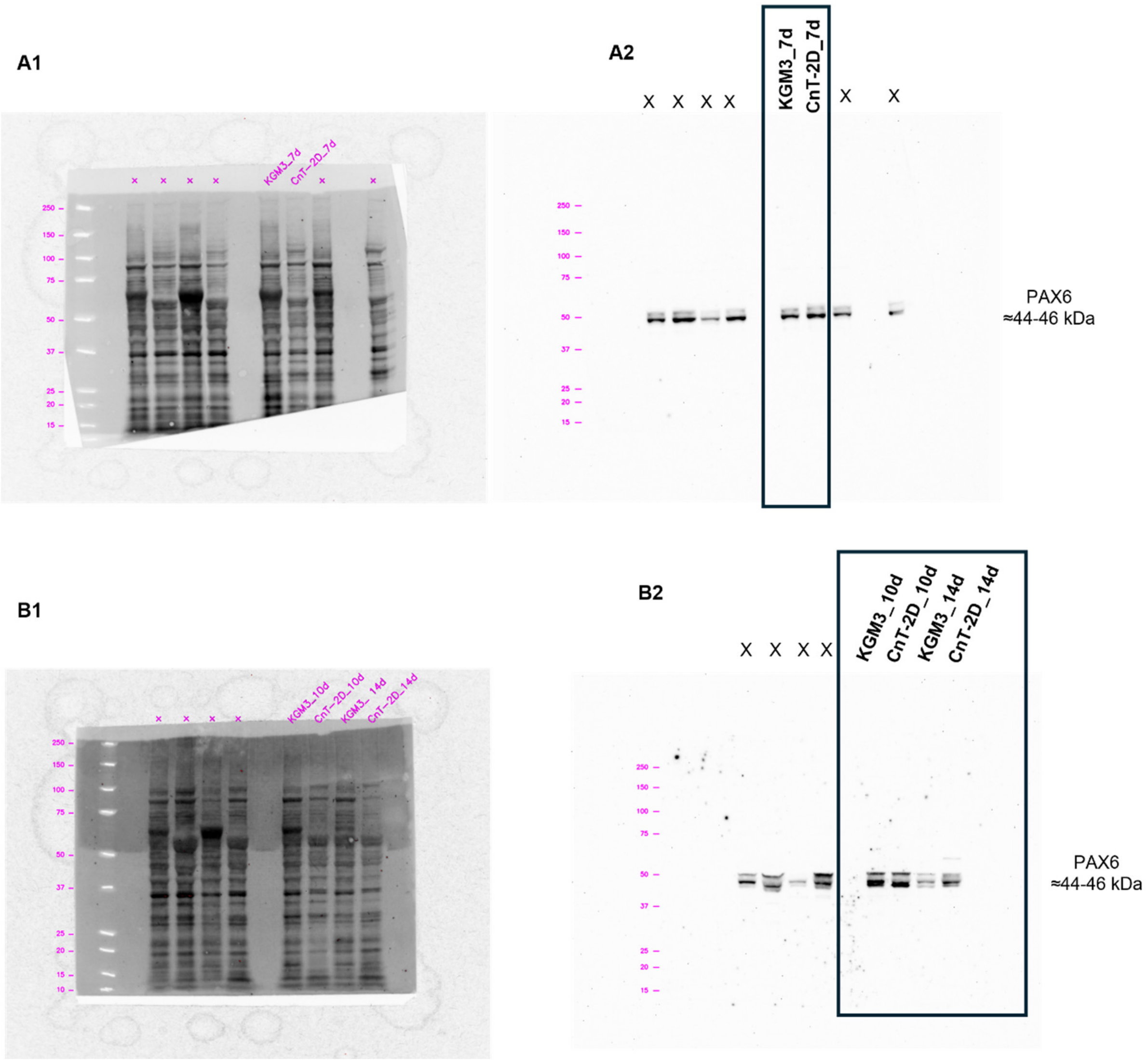


**Supplementary Figure S4. PAX6 Western blot images. (A1, B1)** Total protein normalization (TPN) was performed using the Invitrogen™ No-Stain™ Protein Labeling Reagent to ensure equal protein loading. **(A2, B2)** PAX6 protein bands were detected at the expected molecular weight of approximately 44–46 kDa in primary limbal epithelial cells (pLECs) switched from Keratinocyte Growth Medium (KGM3) to CnT-2D differentiation medium (CnT-2D), or maintained in KGM3, for 7 days **(A2)** and for 10 and 14 days **(B2).**

**Supplementary Figure S5.**

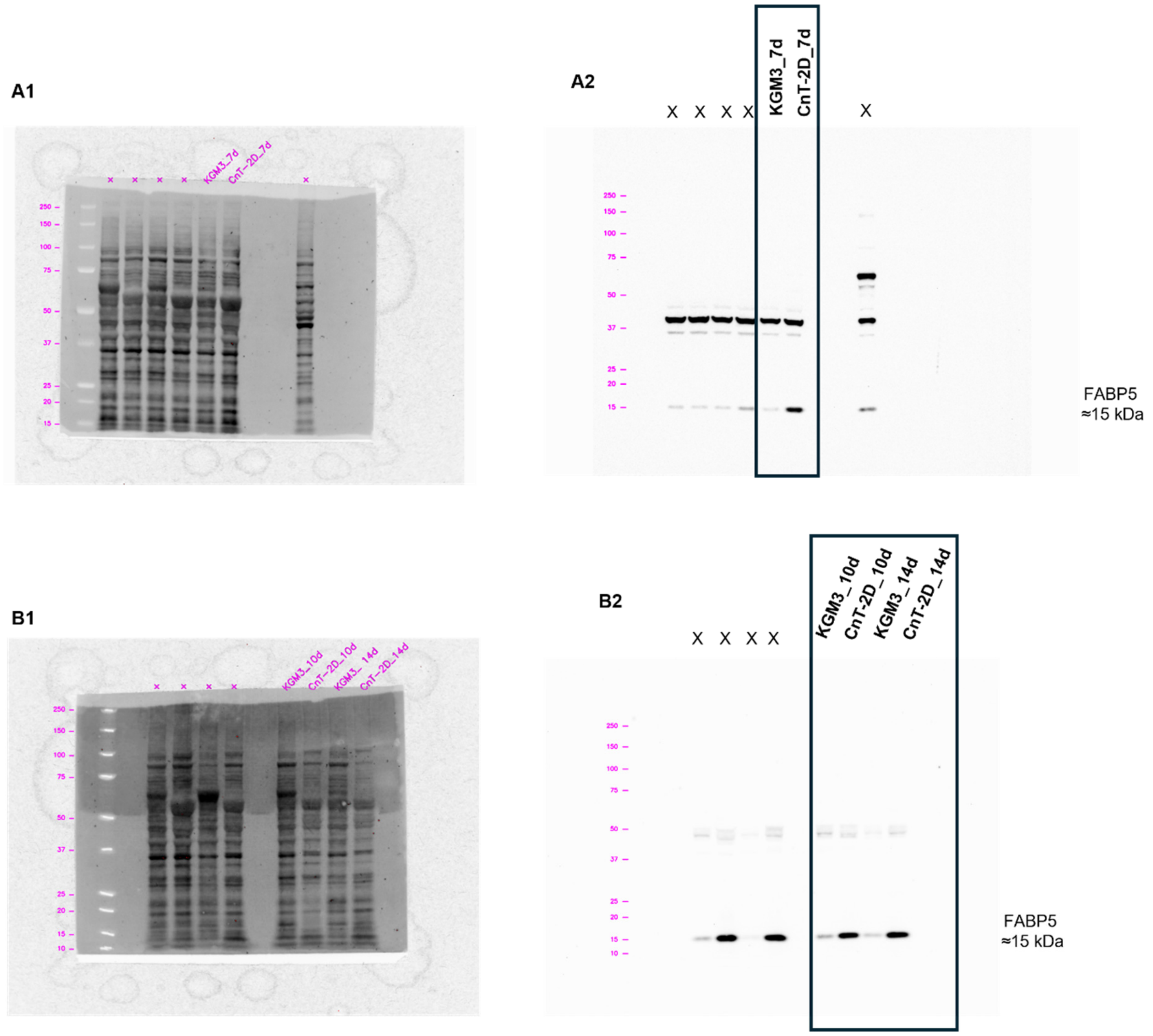


**Supplementary Figure S5. FABP5 Western blot images. (A1, B1)** Total protein normalization (TPN) was performed using the Invitrogen™ No-Stain™ Protein Labeling Reagent to ensure equal protein loading. **(A2, B2)** FABP5 protein bands were detected at the expected molecular weight of approximately 15 kDa in primary limbal epithelial cells (pLECs) switched from Keratinocyte Growth Medium (KGM3) to CnT-2D differentiation medium (CnT-2D), or maintained in KGM3, for 7 days **(A2)** and for 10 and 14 days **(B2).**

**Supplementary Figure S6.**

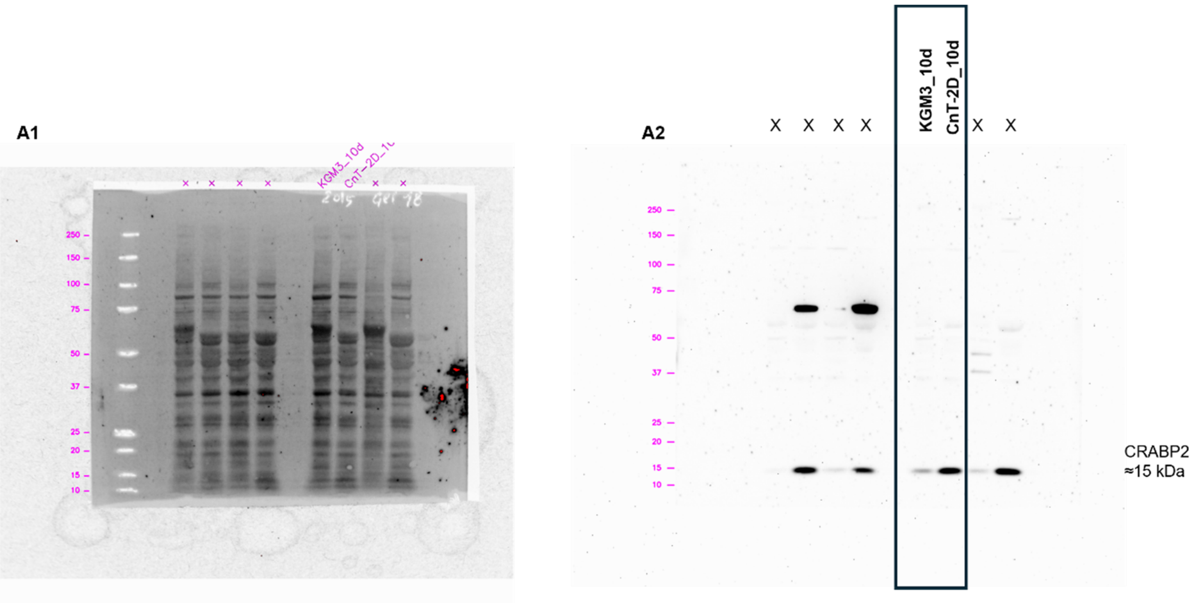


**Supplementary Figure S6. CRABP2 Western blot images. (A1)** Total protein normalization (TPN) was performed using the Invitrogen™ No-Stain™ Protein Labeling Reagent to ensure equal protein loading. **(A2)** CRABP2 protein bands were detected at the expected molecular weight of approximately 15 kDa in primary limbal epithelial cells (pLECs) switched from Keratinocyte Growth Medium (KGM3) to CnT-2D differentiation medium (CnT-2D), or maintained in KGM3, for 10 days.

**Supplementary Figure S7.**

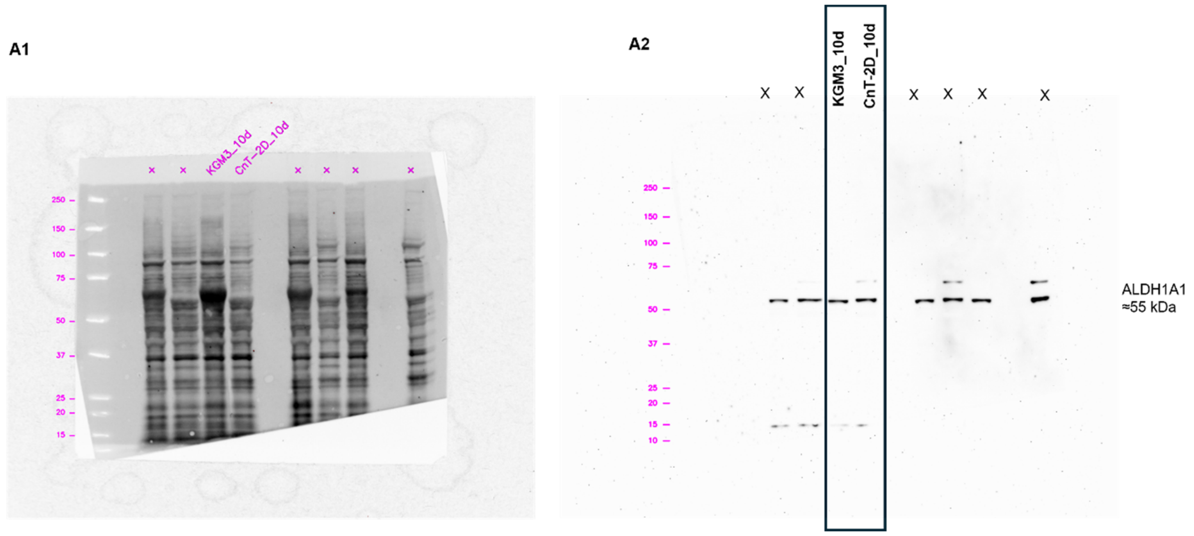


**Supplementary Figure S7. ALDH1A1 Western blot images. (A1)** Total protein normalization (TPN) was performed using the Invitrogen™ No-Stain™ Protein Labeling Reagent to ensure equal protein loading. **(A2)** ALDH1A1 protein bands were detected at the expected molecular weight of approximately 55 kDa in primary limbal epithelial cells (pLECs) switched from Keratinocyte Growth Medium (KGM3) to CnT-2D differentiation medium (CnT-2D), or maintained in KGM3, for 10 days.

**Supplementary Figure S8.**

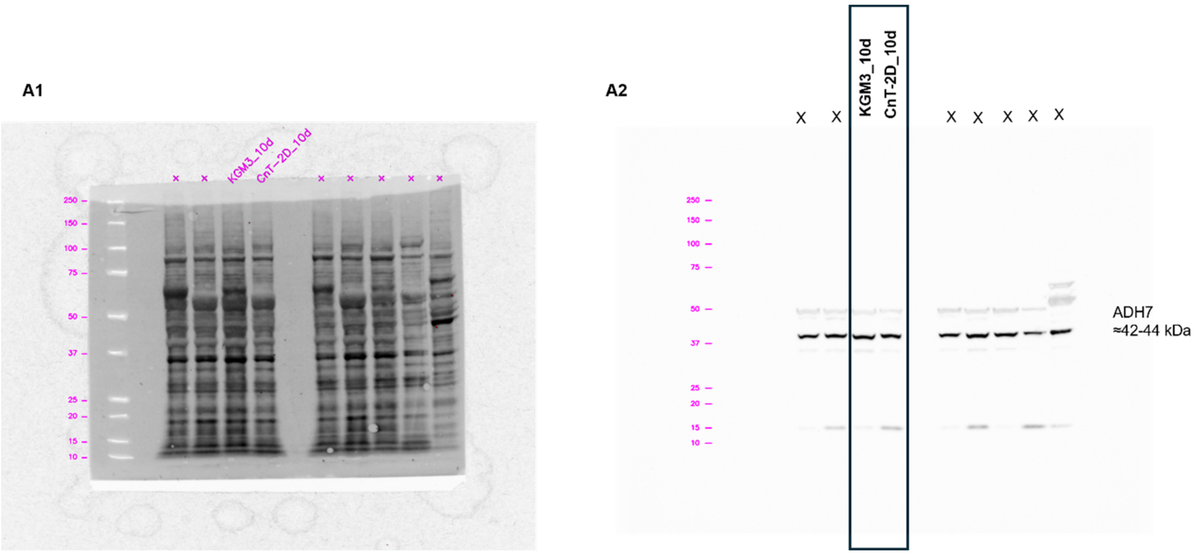


**Supplementary Figure S8. ADH7 Western blot images. (A1)** Total protein normalization (TPN) was performed using the Invitrogen™ No-Stain™ Protein Labeling Reagent to ensure equal protein loading. **(A2)** ADH7 protein bands were detected at the expected molecular weight of approximately 42–44 kDa in primary limbal epithelial cells (pLECs) switched from Keratinocyte Growth Medium (KGM3) to CnT-2D differentiation medium (CnT-2D), or maintained in KGM3, for 10 days.
